# Coleman v. Coldwell

**Published:** 1899-04-22

**Authors:** 


### The Hospital

A JOURNAL OF

The Medical Sciences and Hospital Administration.

VOL. XXVI.—NO. 656.] EDITED BY SIR HENRY BURDETT, K.C.B., AND SOLOMON C. SMITH, M.D., M.R.C.P. [APRIL 22, 1899.

### COLEMAN V. COLDWELL.

THE case of Coleman *versus* Coldwell and Co., tried last week in the Queen's Bench Division, before Mr. Justice Phillimore, appears to show that the "counter prescribing" druggist is not the chartered libertine that he has been supposed to be, and that he is liable to be cast in damages for the injurious effects of the "remedies" which he "prescribes," or, more correctly, of the edge tools with which he plays. The plaintiff was a little girl six years of age, suing by her father as her next friend; and the newspaper report gave no information concerning the social or educational position of the family. The plaintiff had a sore place on her cheek, which her mother supposed to be (and which, according to the report, "in fact was") ringworm. The mother went to a shop kept by the defendants at Watford, and asked for something to cure this ringworm. The defendants' assistant, who did not appear to have seen the child, and who would presumably have been no wiser if he had seen her, sold the mother a box of the ointment of nitrate of mercury of the British Pharmacopœia, to be applied to the sore place, and there was a conflict of evidence as to the manner in which the application was directed to be made. The ointment set up a "violent irritation," which became worse after it had been used a second time. The father then went to the defendants' shop for further "advice," but found it closed, and the matter seems to have rested for a few days, when another "chemist" was applied to, and ultimately Dr. Robbins was consulted. By that time the child had a permanent and disfiguring scar upon her face, said to be a consequence of the irritation excited by the ointment. Medical evidence was given, but not reported, although its general effect was stated to be that the ointment was unsuitable to the stage of the disease in which it was used, and that the directions given as to the mode of using it were insufficient. The jury proposed in the first instance to give "the smallest amount of damages that would carry costs," but the Judge refused to receive such a verdict, and one was finally given for £5. The Judge certified for costs on the High Court scale, which, as the trial occupied two days, will practically amount to a very considerable fine upon the defendants.

The Judge, in announcing his decision about costs, said that the case was "of some importance," and both druggists and the medical profession are likely to agree with him in this opinion. It has hitherto been generally supposed that the victim of injurious "counter practice" does but furnish an example of the validity of the maxim *caveat emptor*, and that the druggist who does not hold himself out to be a qualified medical practitioner is at liberty to sell sixpennyworth of anything to anyone who may ask his advice. The theory has been that the sick person could go into a druggist's shop and could say to him, as he might say to any casual acquaintance in the street, "What is a good thing for jaundice, or for gout, or for indigestion?" and, on receiving a reply, could continue the conversation by directing that some of the alleged remedy should be prepared for him. The seller has been supposed only to express an individual opinion, which the buyer might take for what it was worth, and might act upon or disregard as he pleased, in either case purely at his own personal risk. The case of Coleman seems to establish a new and very salutary principle, and one which, if it be applied whenever possible, will have a marked tendency to reduce "counter practice" within comparatively narrow limits. The average druggist will be less willing to sell his sixpennyworths when he realises that he will be answerable in pocket for any injury which they may inflict. It is to be hoped that the Medical Defence Union, or some analogous body, will take prompt measures to "rub in" the lesson which the case is calculated to convey; and it cannot be doubted that materials sufficient for the purpose might speedily be obtained from coroners exercising jurisdiction in crowded communities, or from medical practitioners whose work lies much among the artisan and operative classes. At a time when the Medical Council, after some years of warning, has at last taken steps finally to extinguish the employment by medical men of unqualified assistants in any really professional capacity, it is manifest that the public should also be protected from another and even more insidious form of the same evil. The case of Coleman appears to be one of a numerous class, in which the powers of the existing law, if fully employed, are quite sufficient to produce effects which "reformers" are apt to seek from fresh legislative enactments.

